# An epoxydinaphthalene monomer for the synthesis of a poly(aryl ether sulfone) and a poly(aryl ether ketone) with enhanced performance for membrane gas separations

**DOI:** 10.1039/d6py00312e

**Published:** 2026-08-17

**Authors:** Yuancheng Liu, John M. Tobin, Gary. S. Nichol, Dominic Taylor, Jiyao Huang, Carmen Rizzuto, Johannes C. Jansen, Alessio Fuoco, Neil B. McKeown

**Affiliations:** a EaStChem, School of Chemistry, University of Edinburgh David Brewster Road Edinburgh EH9 3FJ UK neil.mckeown@ed.ac.uk; b National Research Council of Italy – Institute on Membrane Technology “Enrico Drioli” (CNR-ITM) Via P. Bucci 17/C 87036 Rende CS Italy

## Abstract

A novel monomer based on epoxydinaphthalene (EDN), a highly rigid bridged-bicyclic structural unit, was prepared using a high yielding reaction between 2,7-hydroxynaphthalene and the hydrate of 1,8-naphthalene dialdehyde. This monomer was used to make a poly(aryl ether ketone) and poly(aryl ether sulfone), EDN-PAEK and EDN-PAES, *via* efficient aryl ether bond formation, by reaction with cheaply available co-monomers. Both EDN-PAEK and EDN-PAES proved readily soluble in organic solvents allowing structural characterization and ready fabrication of robust self-standing films. Gas permeability measurements for He, H_2_, O_2_, N_2_, CO_2_ and CH_4_ indicated that these polymers provide a good trade-off between high selectivity and modest permeability relative to previously published data for structurally related polyketones or polysulfones.

## Abstract 2

Polysulfone and polyketone polymers, derived from an epoxydinaphthalene-based monomer, demonstrate useful performance for use as gas separation membranes.

## Graphical abstract

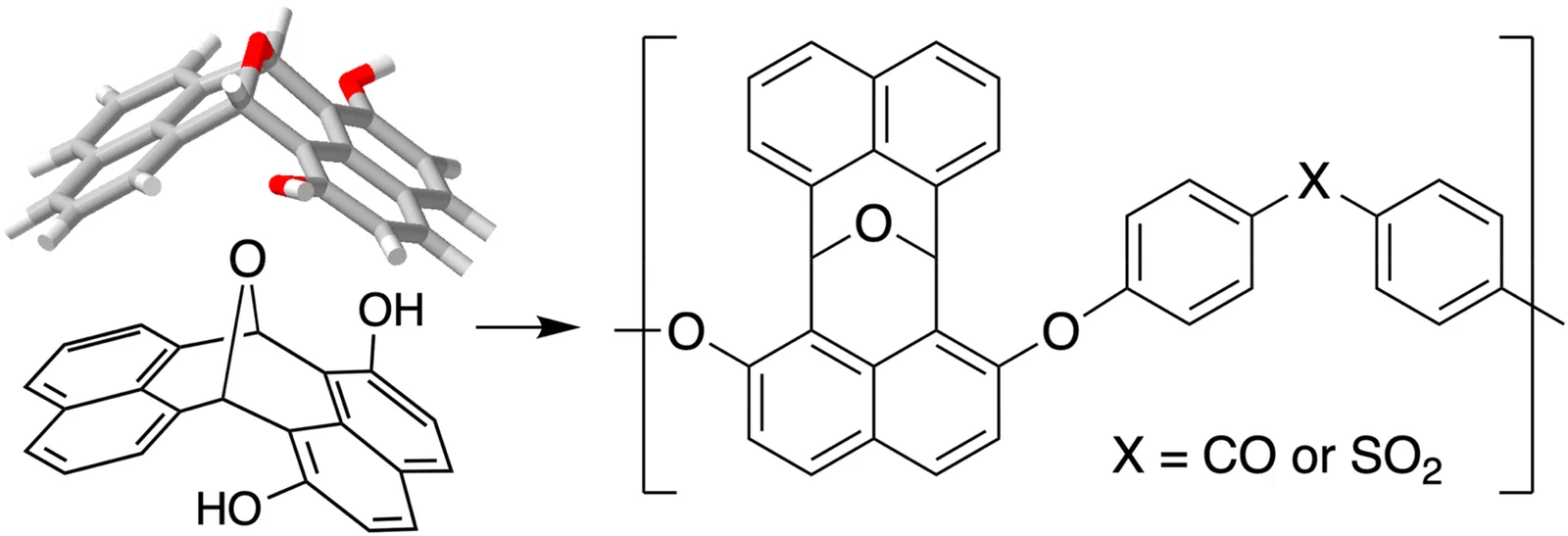

## Introduction

In the chemical industry, thermal separation processes such as distillation are highly energy-intensive, accounting for roughly 10–15% of the world's total energy consumption.^1^ As a more energy-efficient alternative to traditional separation techniques, polymer membrane-based separations are particularly attractive for industrial applications such as natural gas and biogas upgrading (CO_2_/CH_4_), oxygen and nitrogen enrichment of air (O_2_/N_2_), and hydrogen recovery from ammonia purge gases (H_2_/N_2_).^2–4^ For gas separation membranes, good selectivity and high permeability are required to enhance both efficiency and productivity. However, polymeric membranes suffer from the well-known empirical trade-off between permeability (*P*_a_) and ideal selectivity (*P*_a_/*P*_b_) for common gas pairs, with highly permeable polymers exhibiting low selectivity and *vice versa*.^5^ This empirical trade-off was analysed quantitatively by Robeson, who defined upper bounds of permeability *versus* selectivity for most gas pairs.^6,7^ Based on the structure of the polymers that defined these upper bounds, Freeman suggested that their size-sieving property arises from their rigid backbones and therefore, further increasing chain rigidity and inter-chain spacing would enhance the polymer's performance as a gas separation membrane.^8^ Subsequently, extensive research has been devoted to developing polymers that combine high permeability with high selectivity. Ladder polymers such as Polymers of Intrinsic Microporosity (PIMs) with their contorted and rigid macromolecular chain structures, which inhibit efficient packing, confirm to these design criteria.^9^ PIMs have emerged as promising materials for gas separation membranes due to their excellent solution processability, high surface area and high fractional free volume (FFV).^10^ In particular, the gas permeability data of PIMs with rigid bridged bicyclic units such as triptycene,^11,12^ benzotriptycene,^13,14^ and Tröger's base^15^ have resulted in the redefinition of the upper bounds for several commercially important gas pairs.^14,16^ The introduction of these rigid bridged bicyclic structural units provides a contortion site and thus delivers polymers with high free volume, leading to exceptional permeability combined with high selectivity.^17,18^

For several decades, PAEK and PAES polymers have been of interest for making separation membranes due to their excellent thermal stability, high mechanical strength and good solvent resistance.^19^ Widely available PAEK materials, which are used primarily as engineering plastics (*i.e.* those derived from hydroquinone) possess a semi-crystalline morphology, which due to low fractional free volume result in very low gas permeability.^20–22^ Therefore these materials have found more use as supports or organic solvent nanofiltration membranes.^23,24^ By using more complex bisphenol monomers, such as Bisphenol A,^25^*t*-butyl substituted hydroquinone,^22^ fluorinated Bisphenol-F,^26^ and a cardo-lactone bisphenol,^27^ fully amorphous PAEK polymers can be obtained with modest gas permeability. PAES materials derived from diverse bisphenol precursors have been more intensively studied for potential use in gas separations,^28,29^ prompted by the early commercial uptake of membranes using hollow fibres made from the PAES derived from Bisphenol A.^30^ Despite the large body of published research on the gas permeability of PAEK and PAES polymers, these two classes of polymer generally provide data that lie below the 1991 upper bounds with none used by Robeson to define the 1991 or 2008 upper bounds.^6,7^ In addition, few PAEK and PAES polymers have been prepared from a monomer containing a bridged bicyclic monomer,^31^ which based upon the results obtained for PIMs, might be anticipated to improve their performance.

**Fig. 1:**
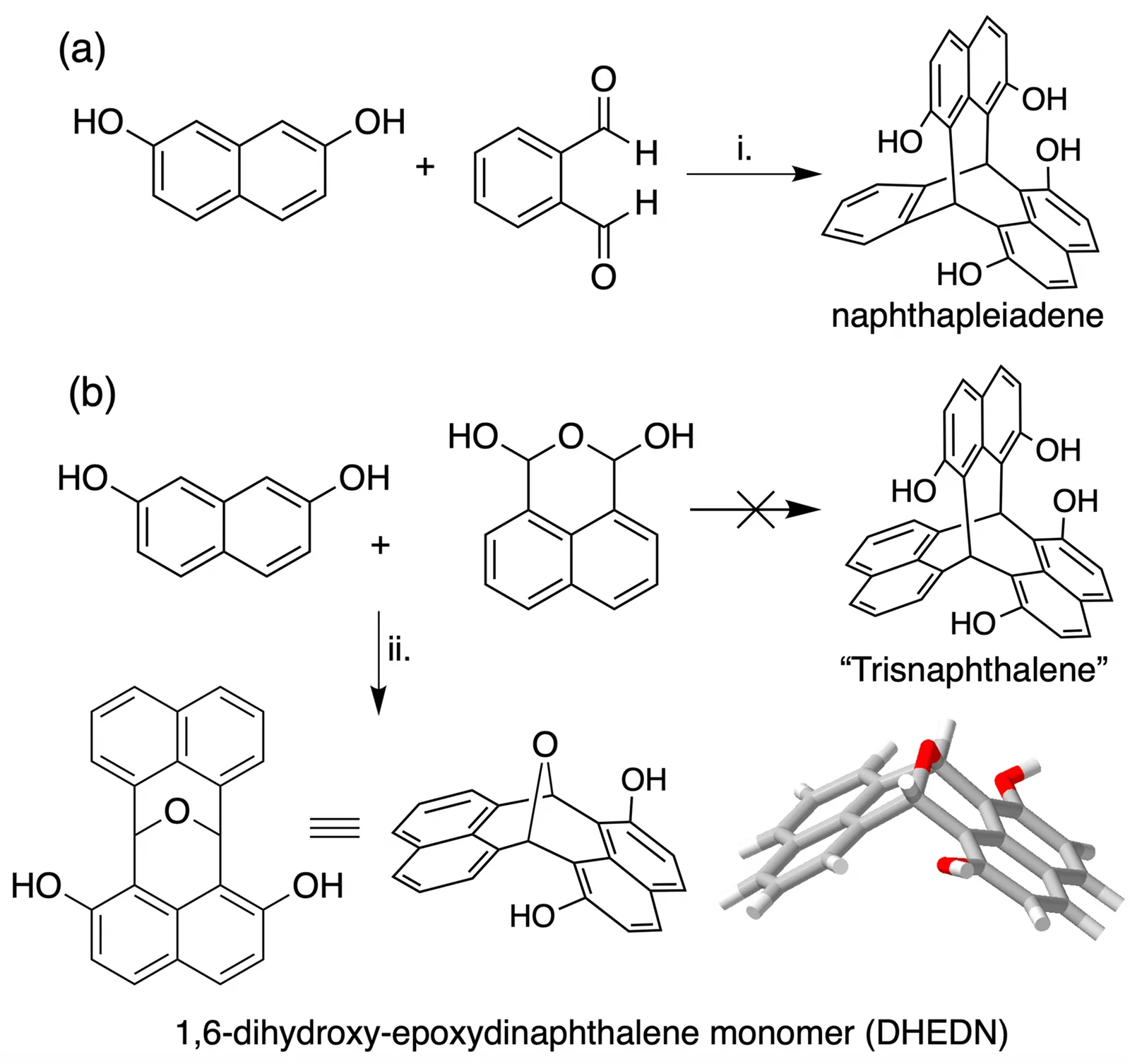
The synthesis of (a) tetrahydroxy-naphthapleiadene, reagents and conditions: (i) conc. aq. HCl, MeOH, reflux 1 h. (b) The synthesis and structure from single crystal XRD analysis of the dihydroxyepoxydinaphthalene monomer DHEDN during the attempted synthesis of tetrahydroxy-trisnaphthalene. (ii) Reagents and conditions: conc. aq. HCl, AcOH, reflux, 24 h.

As part of our continuing research programme on identifying useful and readily prepared triptycene-like structural units for making high performance gas separation membranes, we recently described the formation of tetrahydroxynaphthapleiadene by the simple acid mediated reaction between phthalaldehyde and 2,7-dihydroxynaphthalene (Fig. 1a).^32^ Hence, it was anticipated that a similar reaction between 1,8-naphthalenedialdehyde and 2,7-dihydroxynaphthalene might produce a bridged-bicyclic monomer based on the presently unknown trisnaphthalene structural unit. Trisnaphthalene, a triptycene-like bridged bicyclic compound with three naphthalene rings fused *via* their *peri* (1,8) sites to two bridgehead carbons, has been proposed as the scaffold for producing interesting hypothetical diradical species.^33^ Instead, we obtained a high yield of 7,14-epoxy-1,6-dihydroxy-7*H*,14*H*-cycloocta[1,2,3-*de*:5,6,7-*d*′*e*′]dinaphthalene, henceforth abbreviated to dihydroxyepoxydinaphthalene (DHEDN) (Fig. 1b). The in-built hydroxyl functionality of DHEDN, combined with the ease and efficiency of its synthesis, suggested its use as a monomer for making aromatic polymers using conventional aryl ether formation. Here we report the synthesis and properties of a DHEDN-based polyarylether ketone (EDN-PAEK) and polyarylether sulfone (EDN-PAES) for which its rigid bridged bicyclic structure provides enhanced gas permeability/selectivity over PAES/PAEK polymers derived from more conventional monomer precursors.

## Results

### Synthesis of epoxydinaphthalene-based monomer

It was found that the attempted isolation of the key precursor 1,8-naphthalenedialdehyde, from the oxidation of acenaphthene diol, was difficult and greatly reduced the yield. Instead, the cyclic hydrate of 1,8-naphthalenedialdehyde (1*H*,3*H*-naphtho[1,8-*cd*]pyran-1,3-diol; Fig. 1) was used directly for the reaction with 2,7-dihydroxynaphthalene, which proceeded smoothly using conc. aq. HCl in refluxing acetic acid as solvent (see SI for details). On the addition of water, the DHEDN monomer could be isolated as an off-white solid in 93% yield. Confirmation of the structure of DHEDN was achieved using ^1^H and ^13^C NMR, high resolution mass spectrometry and single crystal X-ray diffraction (CCDC 2539757; Fig. 1). The ^1^H NMR shows two distinctive singlet peaks corresponding to the bridgehead protons at 6.56 ppm and to the –OH groups at 10.02 ppm (SI Fig. S1). Previously, similar epoxydinaphthalene derivatives have been obtained, also serendipitously, from the attempted Vilsmeier–Haack formylation of 1,3,6,8-tetramethoxynaphthalene^34^ and the attempted acylation of the “proton sponge” 1,8-bis(dimethylamino)naphthalene with trifluoroacetic anhydride.^35^ The DHEDN monomer appears to be very stable and unreactive towards further reaction as demonstrated by our failed attempts to achieve ring-opening nucleophilic additions even under superacidic conditions (*i.e.* using trifluoromethyl sulfonic acid).

### Synthesis and characterization of epoxydinaphthalene-based polymers

**Fig. 2:**
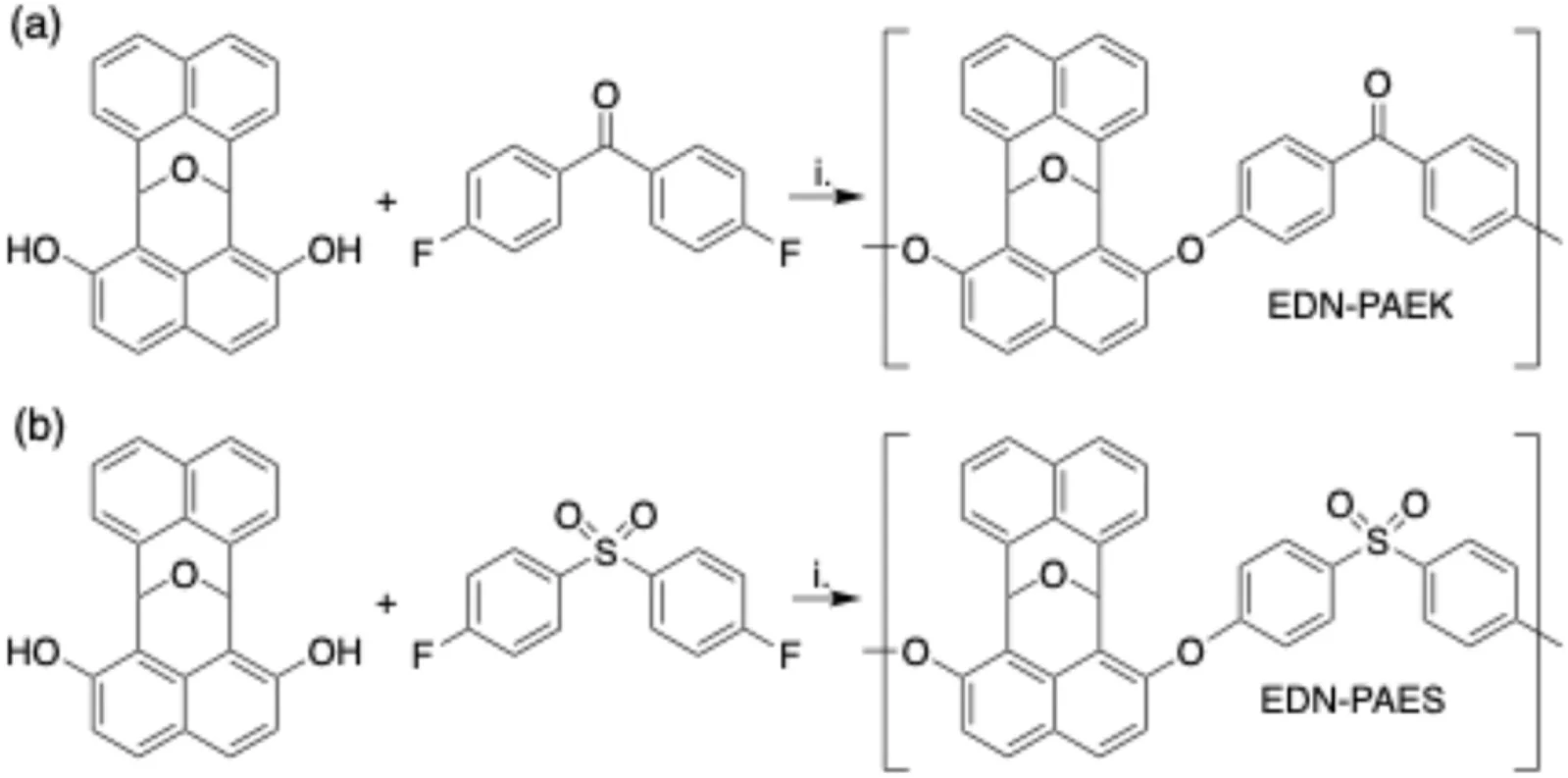
The synthesis of (a) epoxydinaphthalene-based poly(aryl ether ketone) EDN-PAEK and (b) epoxydinaphthalene-based poly(aryl ether sulfone) EDN-PAES; reagents and conditions: (i) K_2_CO_3_, DMAc/toluene, reflux 12 h.

**Fig. 3:**
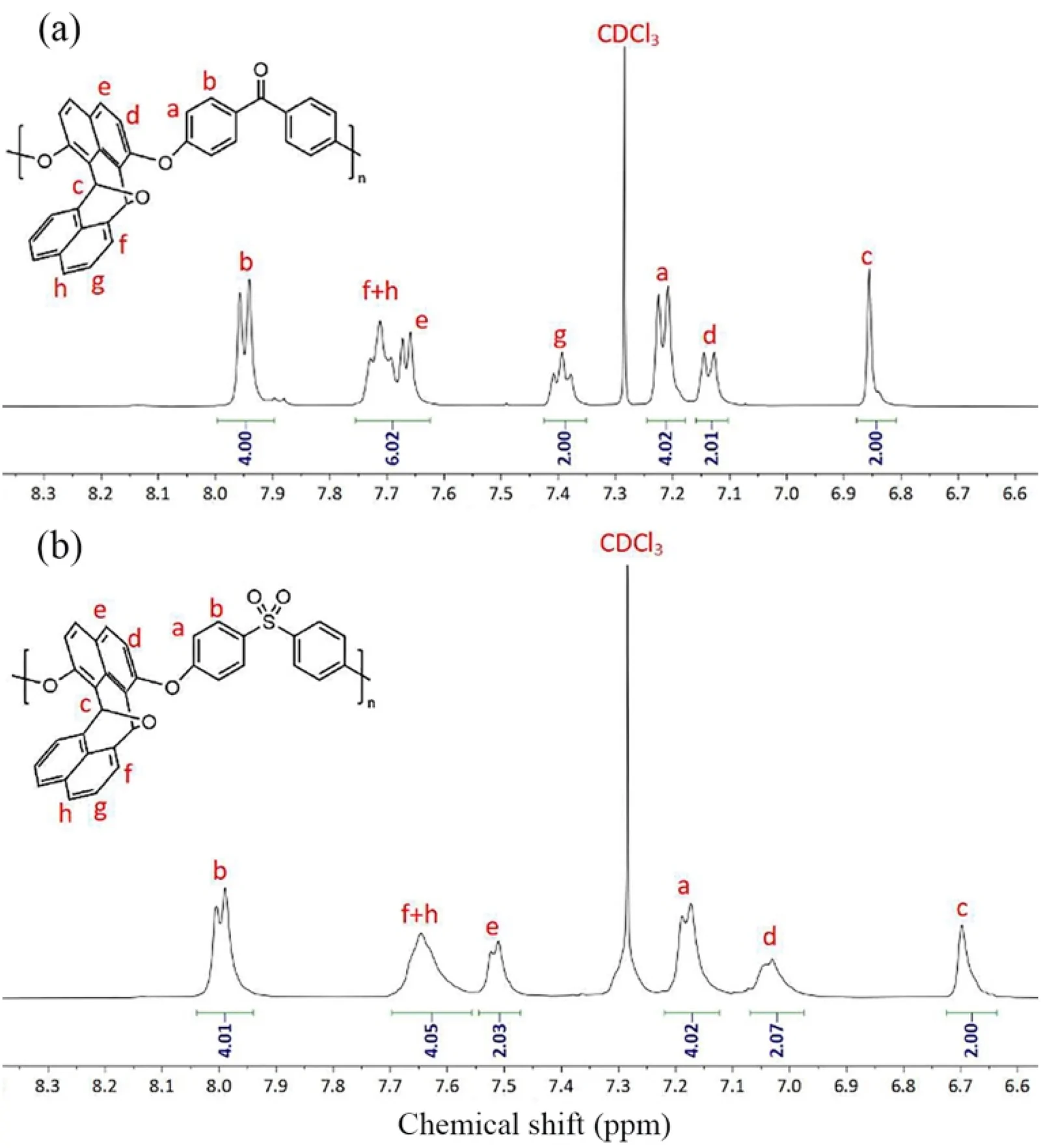
^1^H NMR spectra of (a) EDN-PAEK and (b) EDN-PAES with suggested peak assignment. The anticipated triplet for hydrogen “g” of EDN-PAES is obstructed partially by the peak from the CDCl_3_ solvent.

The DHEDN monomer was used for the synthesis of a poly(arylether ketone) and a poly(aryl ether sulfone) by reaction with commercially available 4,4′-difluorobenzophenone and 4,4′-difluorodiphenyl sulfone, respectively, using well established reaction conditions for aryl ether formation *via* aromatic nucleophilic substitution (Fig. 2). Purification was achieved by reprecipitation from chloroform solution into methanol. The EDN-PAEK and EDN-PAES polymers were characterized by FTIR (SI Fig. S2) and show characteristic peaks at 1655 cm^−1^, corresponds to C=O stretching, and 1105 cm^−1^, attributed to S=O stretching, respectively, in addition to bands expected for aryl ether polymers at 1225 cm^−1^ (aromatic C–O–C stretching). Both EDN-PAEK and EDN-PAES have good solubility in organic solvents such as NMP, DMAc and chloroform, allowing their characterisation by ^1^H NMR analysis (Fig. 3). As expected, the NMR spectra are similar for both polymers with the bridgehead methine hydrogen giving a peak at 6.85 ppm for EDN-PAEK and 6.70 ppm for EDN-PAES, with the aromatic protons (Ar–H) appearing in the range of 7.0–8.0 ppm. The good solubility of EDN-PAEK and EDN-PAES in chloroform also facilitated characterisation by Gel Permeation Chromatography (GPC), with values for *M*_n_ = 16.7 × 10^3^ g mol^−1^ (*M*_w_/*M*_n_ = 2.8) and *M*_n_ = 18.2 × 10^3^ g mol^−1^ (*M*_w_/*M*_n_ = 1.5) g mol^−1^, respectively, *versus* polystyrene standards (SI Fig. S3). It is anticipated that much higher molecular mass could be obtained by optimising the polymerisation reactions but this was unnecessary for the present study as the resulting polymers gave robust self-standing films. Thermal gravimetric analysis (TGA) showed no degradation below 350 °C (SI Fig. S4) and differential scanning calorimetry (DSC) indicated no clear thermal transitions up to 300 °C (SI Fig. S5). Powder X-ray diffraction confirms that both samples are amorphous as expected for glassy polymers (SI Fig. S6). Nitrogen (N_2_) adsorption at 77 K (SI Fig. S7) indicated that powdered samples of both polymers are non-porous with only a small up-take at low pressure for EDN-PAES, allowing an apparent BET surface area of 39 m^2^ g^−1^ to be calculated whereas EDN-PAEK showed no N_2_ uptake at low pressure. Carbon dioxide (CO_2_) adsorption at 1 bar and 273 K (SI Fig. S8) was greater for EDN-PAES (∼0.8 mmol g^−1^) than for ED-PAEK (0.6 mmol g^−1^) but these values are much lower than those typically obtained for PIMs (1.5–2.5 mmol g^−1^).^36^

### Gas transport properties of EDN-PAEK and EDN-PAES

The pure gas permeabilities and ideal selectivities for EDN-PAEK and EDN-PAES, as measured from robust self-standing films formed by casting from chloroform solutions (SI Fig. S9), are summarised in Table 1. Measurements were made from freshly methanol treated films and the same films after 120 days of aging at ambient conditions. Typically, the gas permeability of aged films of glassy polymers is lower due to physical aging (*i.e.* loss of free volume). For all gases, EDN-PAES proved to be slightly more permeable than EDN-PAEK with the gas permeability order for both polymers being H_2_ > He > CO_2_ > O_2_ > N_2_ > CH_4_. The use of the time-lag method to measure gas permeabilities also provide estimates of diffusivity coefficients, (SI Table S1) and solubility coefficients (SI Table S2) and showed that the higher permeability of EDN-PAES compared to EDN-PAEK is attributed to its higher gas diffusivity coefficients for all gases.

**Table 1:** Gas permeabilities (Barrer) and ideal selectivities of EDN-PAEK and EDN-PAES films freshly treated with methanol and after aging for 120 days measured at 35 °C and 1 bar

	Permeability (Barrer)						Selectivity (—)					
	N_2_	O_2_	CO_2_	CH_4_	H_2_	He	CO_2_/CH_4_	CO_2_/N_2_	O_2_/N_2_	H_2_/N_2_	H_2_/CH_4_	He/N_2_
EDN-PAEK	0.42	3.09	13.3	0.27	34.7	26.8	49.3	31.7	7.4	82.7	128.6	63.7
(120 d)	0.26	1.99	9.54	0.22	26.4	20.7	42.8	36.7	7.65	101	118	80
EDN-PAES	0.76	4.52	25.9	0.68	51.0	38.7	38.1	34.1	5.9	67.1	74.9	50.8
(120 d)	0.42	3.01	18.2	0.44	40.8	33.3	41.8	43.7	7.2	98.2	93.8	80
SBI-PAES^37^	10.8	30.0	90.0	13.4	184	147	6.7	8.3	2.8	17.0	13.7	13.6
Trip-PAES^31^	0.2	1.7	6.4	0.2	27.0	—	37.7	27.8	7.4	117	159	—

The higher gas diffusivity for EDN-PAES could be due to the bulkier sulfone group enhancing the fractional free volume, as seen by the linear correlation with the effective diameter of the gas molecules (SI Fig. S10).^17^ The higher free volume of EDN-PAES is consistent with its greater uptake of CO_2_ relative to that of EDN-PAEK observed for powdered samples at 273 K (SI Fig. S8).

## Discussion

**Fig. 4:**
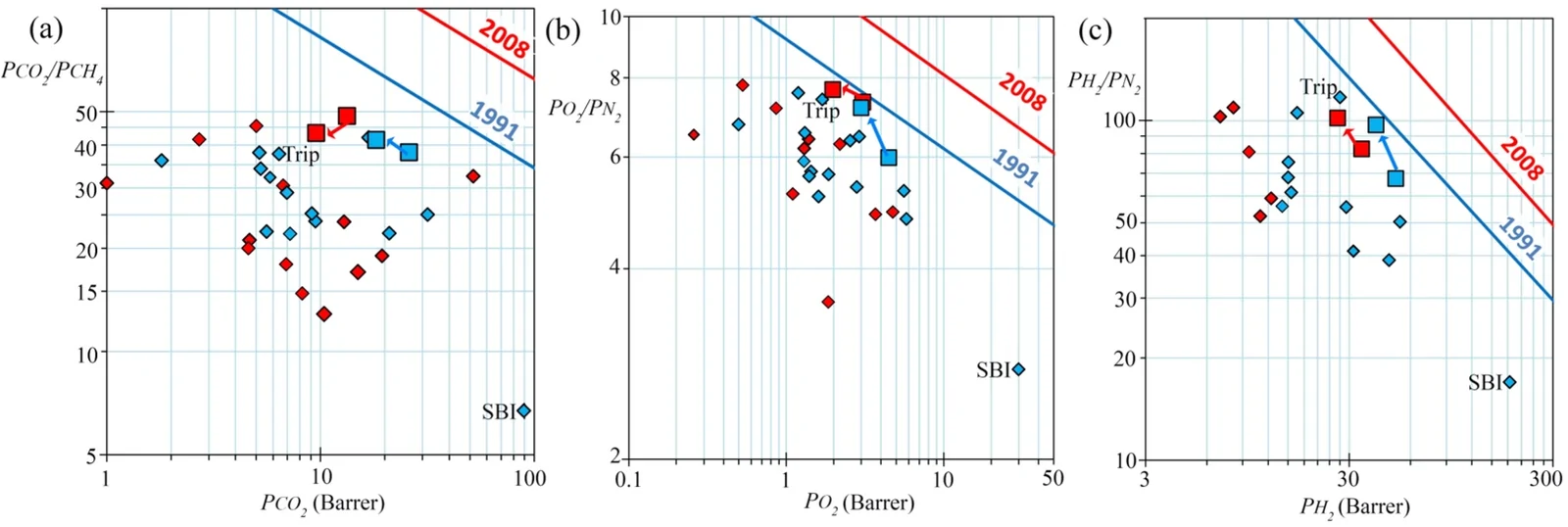
Robeson plots for the (a) CO_2_/CH_4_, (b) O_2_/N_2_, and (c) H_2_/N_2_, gas pairs showing the gas permeability data for films of EDN-PAEK (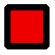) and EDN-PEAS (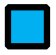), with arrows indicating the change with aging. For comparison, also shown are data reported in the literature for PAEK (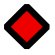)^21,22,25–27,39^ and PAES (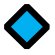)^19,22,28–31,37,40–42^ films. The upper bounds are represented by blue lines (1991) and red lines (2008).^6,7^ The data points for related spirobisindan (SBI) and triptycene (Trip) based PAES polymers are indicated.^31,37^

Both EDN-based polymers exhibit gas permeability and selectivity data that compare favourably to those obtained from the many PAEK and PAES materials reported in the literature. This comparison of performance is best demonstrated by the position of the gas permeability data for EDN-PAEK and EDN-PAES on Robeson plots, for example, those of the O_2_/N_2_, CO_2_/CH_4_ and H_2_/N_2_ gas pairs (Fig. 4). The data points for EDN-PAEK and EDN-PAES lie closer to the 1991 Robeson upper bounds than those for most other PAEK and PAES polymers reported to date (Fig. 4). For example, the CO_2_/CH_4_ data indicate high selectivities for EDN-PAEK and EDN-PAES (PCO_2_/PCH_4_ = 38 and 49, respectively) but with moderate CO_2_ permeability (Fig. 4a). In addition, the data for EDN-PAEK and EDN-PAES (after aging) lie on the O_2_/N_2_ 1991 upper bound (Fig. 4b) indicating a good compromise between high selectivity (PO_2_/PN_2_ > 7.0) and moderate permeability (PO_2_ > 3.0 Barrer). Data for aged EDN-PAES also lie on the 1991 upper bound for H_2_/N_2_ showing enhanced size-selectivity (Fig. 4c). Indeed, comparable performance to EDN-PAEK and EDN-PAES are obtained only from a triptycene-derived PAES (Trip-PAES, Table 1 and Fig. 4)^31^ and fluorinated bisphenol PAEK^26^ with co-polymers of the latter made with sulfonated bisphenol showing further improved performance. The enhanced gas separation performance of EDN-based polymers can be attributed to the highly rigid EDN structural unit, which improves size selectivity by decreasing the diffusivity of larger gas molecules (*i.e.* N_2_ and CH_4_) due to reduced thermal motion helping movement between elements of free volume.^17,18^ Notably, even though the more flexible spirobisindan (SBI) structural unit, which is commonly used for making PIMs,^38^ provides a more permeable PAES, this is at the cost of lower selectivity. Hence the data of SBI-PAES are at a greater distance from the 1991 upper bounds relative to those of EDN-PAEK or EDN-PAES (Fig. 4).^37^

## Conclusions

In summary, a poly(aryl ether ketone) and poly(aryl ether sulfone), EDN-PAEK and EDN-PAES respectively, were prepared using a novel monomer based on the serendipitously prepared highly rigid bridged-bicyclic epoxydinaphthalene structural unit. EDN-PAEK and EDN-PAES proved readily soluble in organic solvents allowing structural characterization and ready fabrication of robust self-standing films. Gas permeability measurements indicated that the permeability and selectivity of both EDN-PAEK and EDN-PAES provide an excellent compromise between high selectivity and moderate permeability for a range of gas pairs including CO_2_/CH_4_, O_2_/N_2_, H_2_/N_2_. Hence, their gas permeability data are placed closer to the 1991 upper bounds for these gas pairs relative to previously published data for PAEK and PAES polymers prepared using more flexible structural units. These data, combined with their ease of synthesis and good film-forming ability, make both EDN-PAEK and EDN-PAES good candidates for further investigation as membrane forming materials.

## Author contributions

Yuancheng Liu: writing – original draft, writing – review & editing, conceptualization, investigation. John Tobin: investigation. Dominic Taylor: investigation. Jiyao Huang: investigation. Carmen Rizzuto: investigation, writing – review & editing. Gary S. Nichol: X-ray crystal structure determination. Alessio Fuoco: writing – review & editing, supervision, investigation, funding acquisition. Johannes C. Jansen: funding acquisition, resources, writing – review & editing, investigation, supervision. Neil B. McKeown: funding acquisition, resources, sonceptualisation, writing – original draft, writing – review & editing, supervision.

## Conflicts of interest

There are no conflicts to declare.

## Supplementary Material

PY-017-D6PY00312E-s001

PY-017-D6PY00312E-s002

## Data Availability

Additional data related to monomer and polymer synthesis, characterisation and gas permeability can be found in the supplementary information (SI). Supplementary information is available. See DOI: https://doi.org/10.1039/d6py00312e. CCDC 2539757 (DHEDN monomer) contains the supplementary crystallographic data for this paper.^43^
