# Supplementary material for: An epoxydinaphthalene monomer for the synthesis of a poly(aryl ether sulfone) and a poly(aryl ether ketone) with enhanced performance for membrane gas separations
Source: Polym Chem. 2026 Aug 17;17(35):3816–21. doi: 10.1039/d6py00312e (PMC13575820; doi:10.1039/d6py00312e)
Supplement: Supplementary file 1 [file PY-017-D6PY00312E-s001.pdf]

Supplementary Information (SI) for Polymer Chemistry.
This journal is © The Royal Society of Chemistry 2026

An epoxydinaphthalene monomer for the synthesis of a poly(aryl ether sulfone) and a poly(aryl ether ketone) with enhanced performance for membrane gas separations

Yuancheng Liu[a], John Tobin[a], Gary S. Nichol[a], Dominic Taylor[a], Jiyao Huang[a], Carmen Rizzuto[b], Johannes C Jansen[b], Alessio Fuoco[b] and Neil B. McKeown*[a]

[a]*EaStChem, School of Chemistry, University of Edinburgh, David Brewster Road, Edinburgh, EH9 3FJ, UK.*

[b]*National Research Council of Italy - Institute on Membrane Technology "Enrico Drioli" (CNR-ITM), Via P. Bucci, 17/C, 87036 Rende (CS), Italy.*

Corresponding author
**Neil B. McKeown E-mail: neil.mckeown@ed.ac.uk*

.

Supplemental information

1. Materials

1.1 Synthesis of EDN monomer

The synthesis of monomer 7,14-epoxy-7H,14H-cycloocta[1,2,3-de:5,6,7-d'e′]dinaphthalene (EDN) is shown in Figure 1 of the main text. A solution of 1,8-naphthalene dialdehyde hydrate (500 mg, 2.47 mmol), 2,7 dihydroxynaphthalene (475 mg, 2.96 mmol) and conc. HCl (1.25 ml) in acetic acid (10 ml) was heated at reflux for 6 h. After cooling to room temperature, the reaction mixture was quenched with water. The resulting precipitate was filtered, washed with water to afford EDN as light purple solid (750 mg, 2.30 mmol, 93%). Mp: >300 °C; FTIR (ν, cm-1): 3300, 3047, 2939, 1638, 1592, 1510, 1266; ^{1}H NMR (500 MHz, DMSO-d6) δ 10.02 (s, 2H, OH), 7.75 (m, 4H, ArH), 7.47 (m, 4H, ArH), 6.91 (d, 2H, ArH), 6.56 (s, 2H, CH); ^{13}C NMR (125 MHz, DMSO-d6) δ 149.65, 136.12, 133.00, 127.79, 127.08, 126.41, 126.16, 124.83, 122.08, 120.64, 115.34, 69.00; HRMS (EI, *m/z*): $[M+H]^+$ calculated for $C_{22}H_{15}O_3$: 327.1015, found: 327.1002. Crystallographic data can be obtained from the Cambridge Crystallographic Data Centre (deposition number CCDC 2539757).

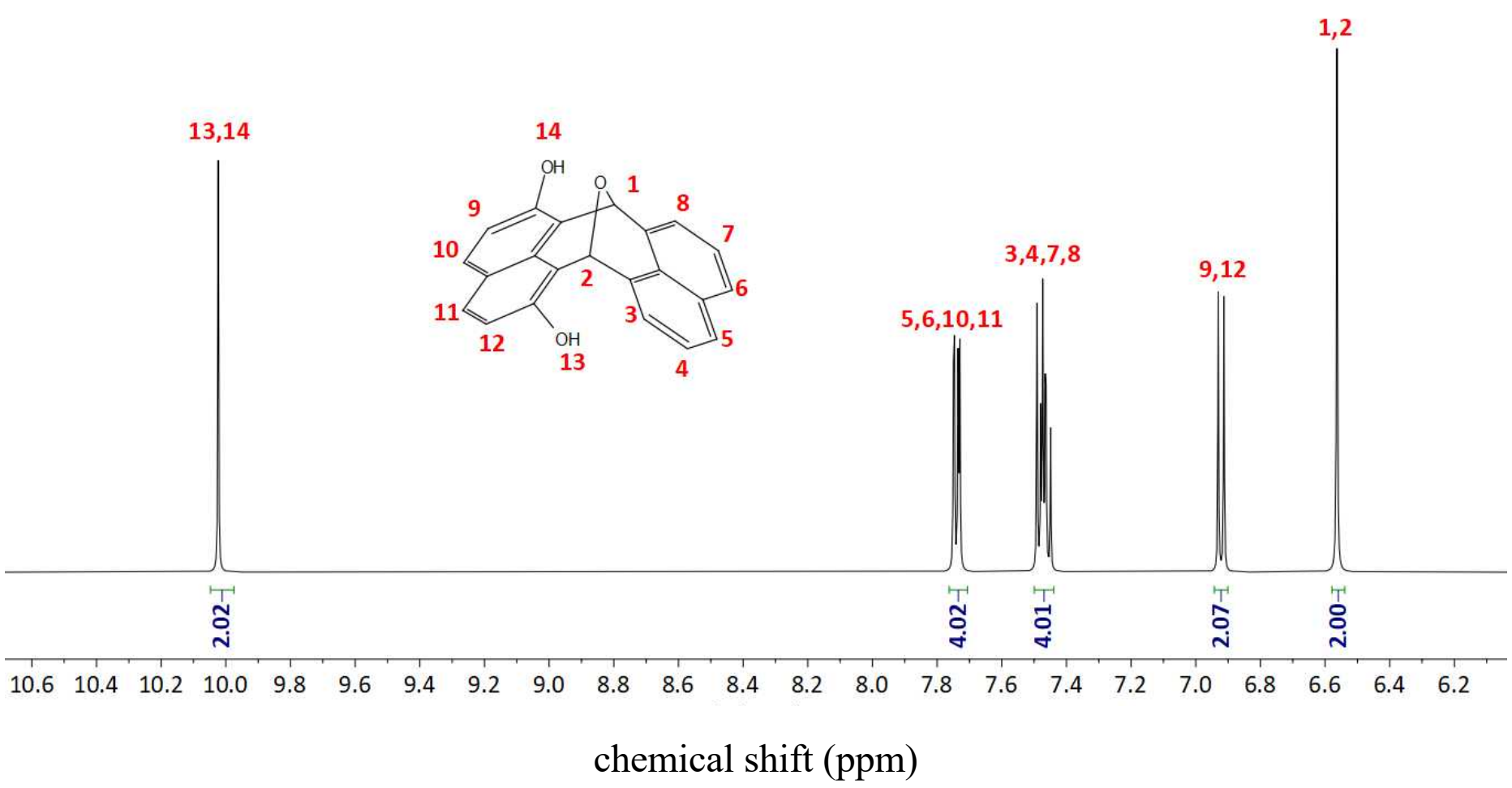

ESI Figure 1. ^{1}H NMR of DHEDN monomer.

1.2 Synthesis of EDN-based polymers

DHEDN monomer (2 g, 6.13 mmol) and 4,4′-difluorobenzophenone (1.34 g, 6.13 mmol) were dissolved in anhydrous *N,N*-dimethylacetamide (DMAc) (20 mL) and anhydrous toluene (5 mL) in a round bottom flask equipped with a Dean-Stark apparatus under nitrogen atmosphere. Potassium carbonate (2,12 g, 15.33 mmol) was added, and the reaction was heated to 140°C to

remove the generated water. After removal, the reaction was heated to reflux (165 °C) for 12 h. The reaction was precipitated in hot water and filtered. The resulting polymer was washed thoroughly with hot water followed by methanol. It was then purified by dissolution in DMAc and reprecipitation from methanol twice, yielding **EDN-PAEK** as a light brown fibrous solid (2.0 g, 65%) after drying in a vacuum oven at 100 °C overnight. FTIR (ν, cm^{-1}): 1655, 1591, 1496, 1225, 1153; ^{1}H NMR (500 MHz, $CDCl_3$) δ 7.94 (d, 4H, ArH), 7.65-7.73 (m, 6H, ArH), 7.39 (t, 2H, ArH), 7.20 (d, 4H, ArH), 7.12 (d, 2H, ArH), 6.85 (s, 2H, CH). Gel Permeation Chromatography: M_n = 16.7 x 10^3 g mol^{-1} (M_w/M_n = 2.8) versus polystyrene standards

The **EDN-PAES** polymer was synthesised using the same procedure. DHEDN monomer (2 g, 6.13 mmol), 4,4′-difluorodiphenyl sulfone (1.56 g, 6.13 mmol), potassium carbonate (2.12 g, 15.33 mmol), dimethylacetamide (DMAc) (20 mL) and anhydrous toluene (5 mL) were combined to afford **EDN-PAES** as a light brown fibrous solid (2.5 g, 75%) after drying in a vacuum oven at 100 °C overnight. FTIR (ν, cm^{-1}): 1628, 1584, 1485, 1230, 1150, 1105; ^{1}H NMR (500 MHz, $CDCl_3$) δ 7.98 (d, 4H, ArH), 7.64 (m, 4H, ArH), 7.51 (d, 2H, ArH), 7.17 (d, 4H, ArH), 7.03 (m, 2H, ArH), 6.69 (s, 2H, CH). Gel Permeation Chromatography: M_n = 18.2 x 10^3 g mol^{-1} (M_w/M_n = 1.5) g mol^{-1}, respectively, versus polystyrene standards

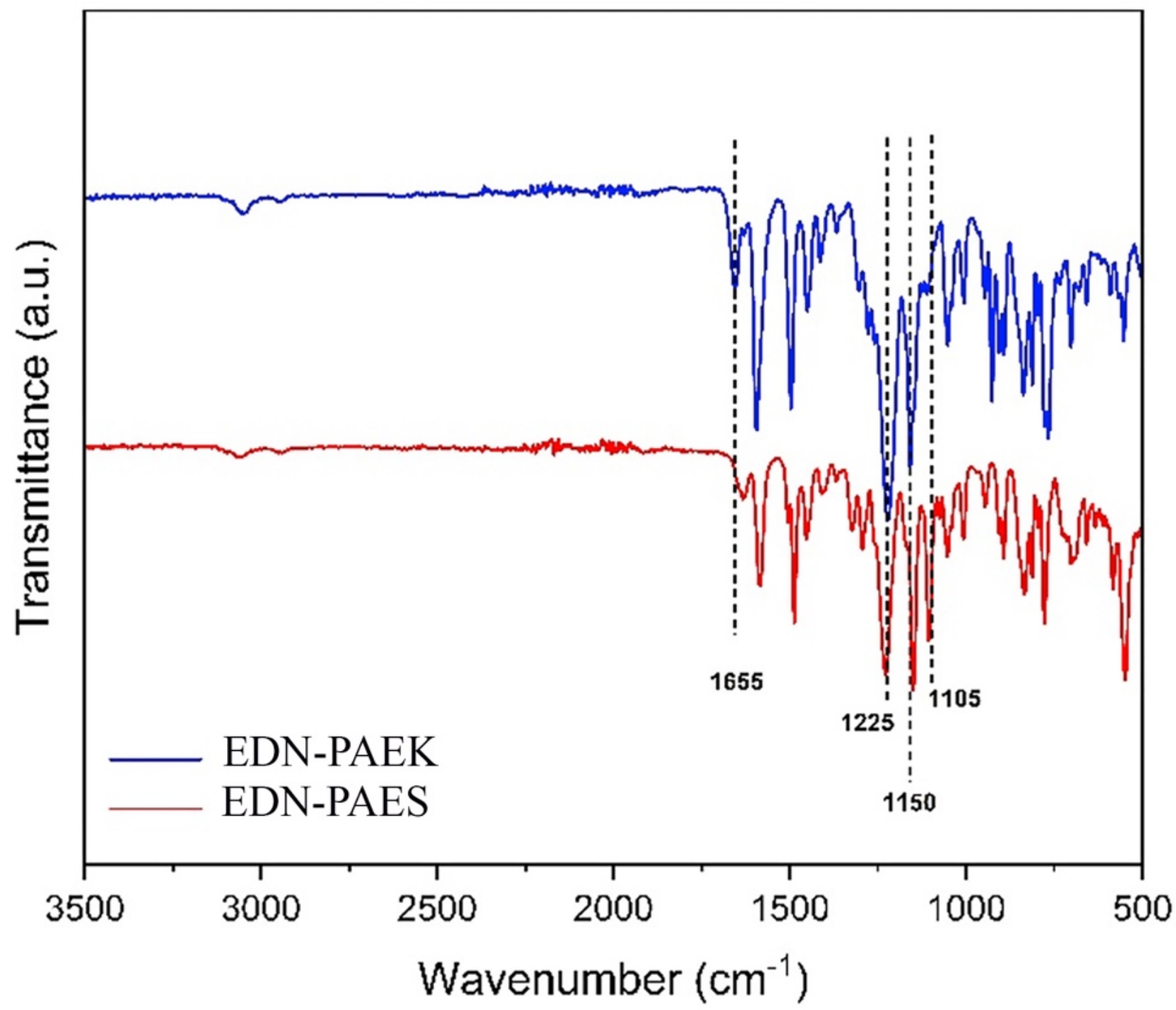

ESI Figure 2. FT-IR spectra of EDN-PAEK and EDN-PAES.

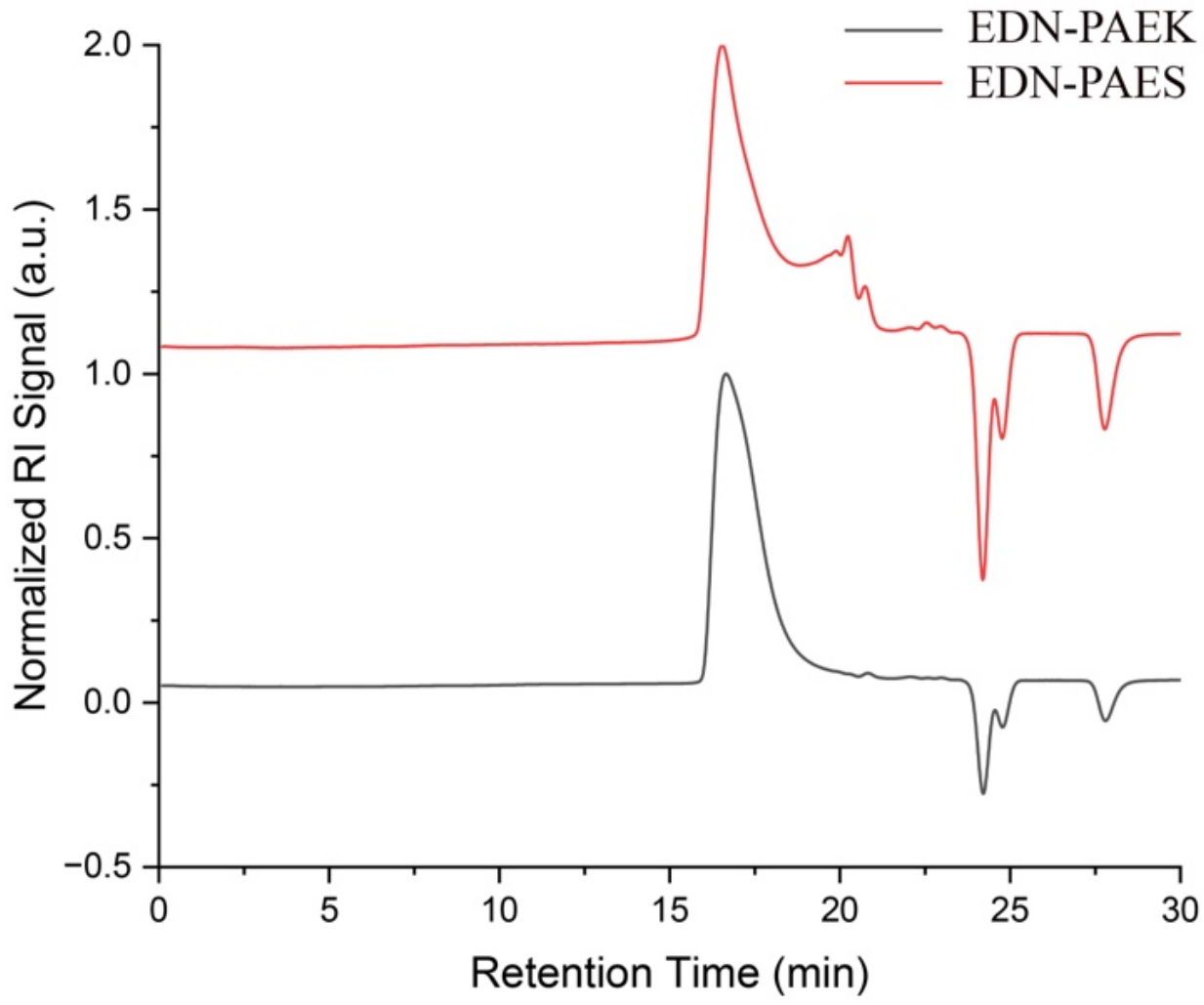

ESI Figure 3. GPC traces of EDN-PAEK and EDN-PAES.

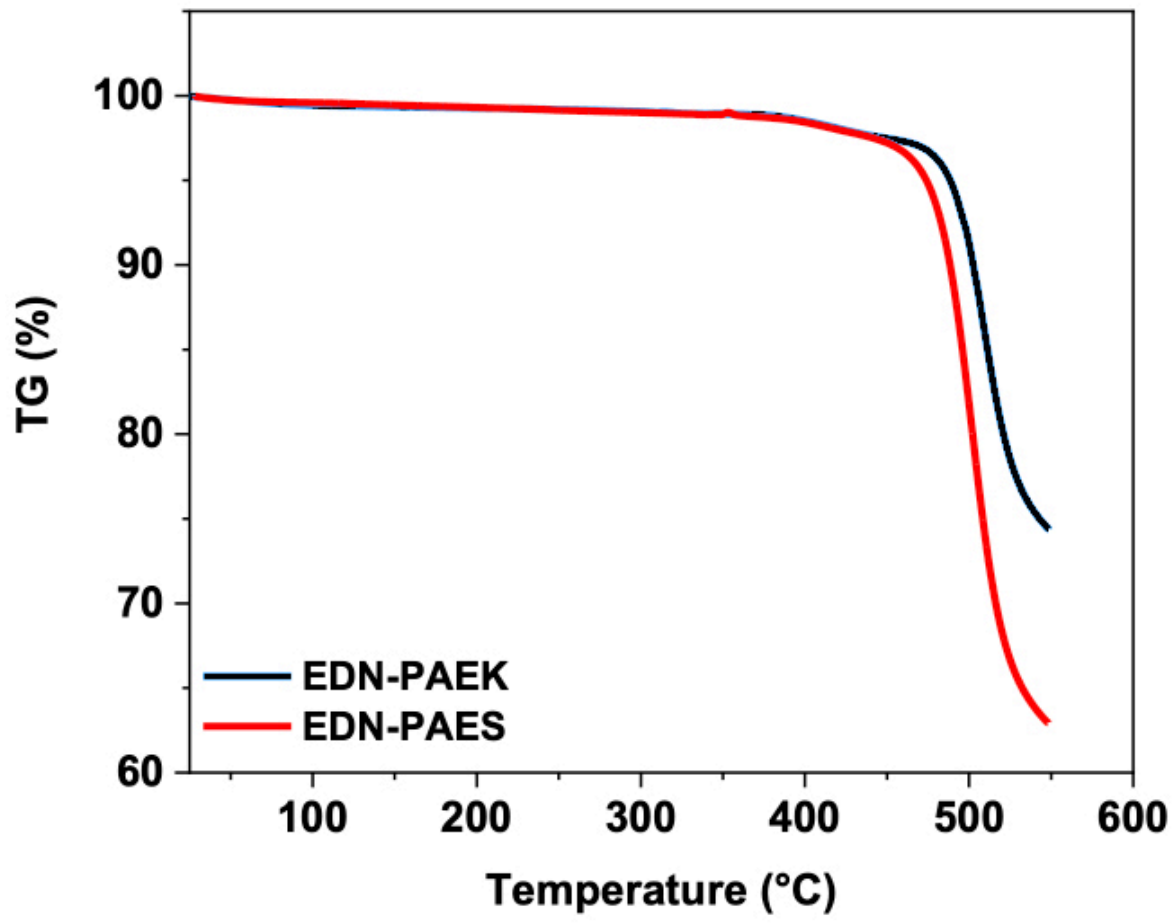

ESI Figure 4. TGA traces of EDN-PAEK and EDN-PAES.

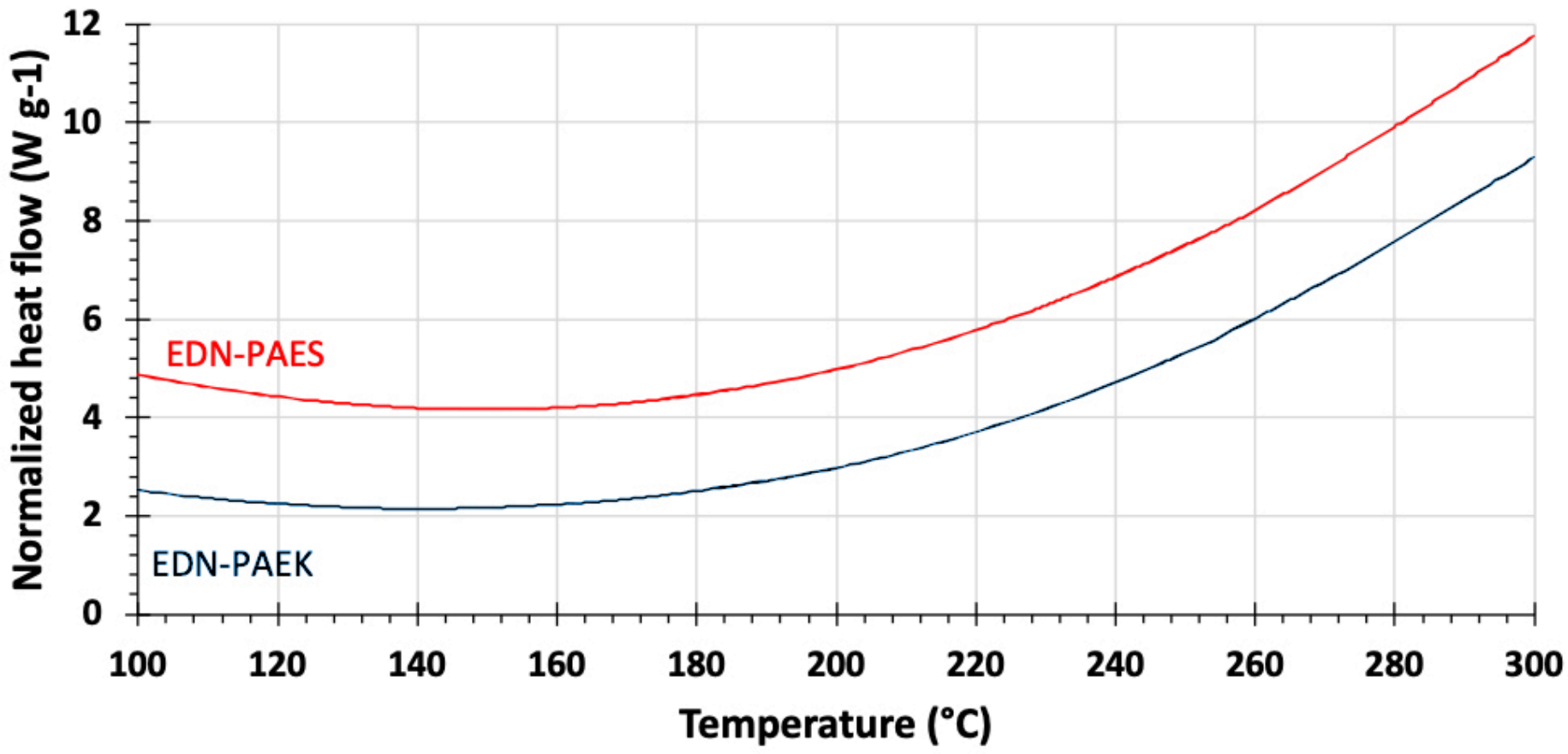

ESI Figure 5. DSC traces of EDN-PAEK and EDN-PAES (second heating run).

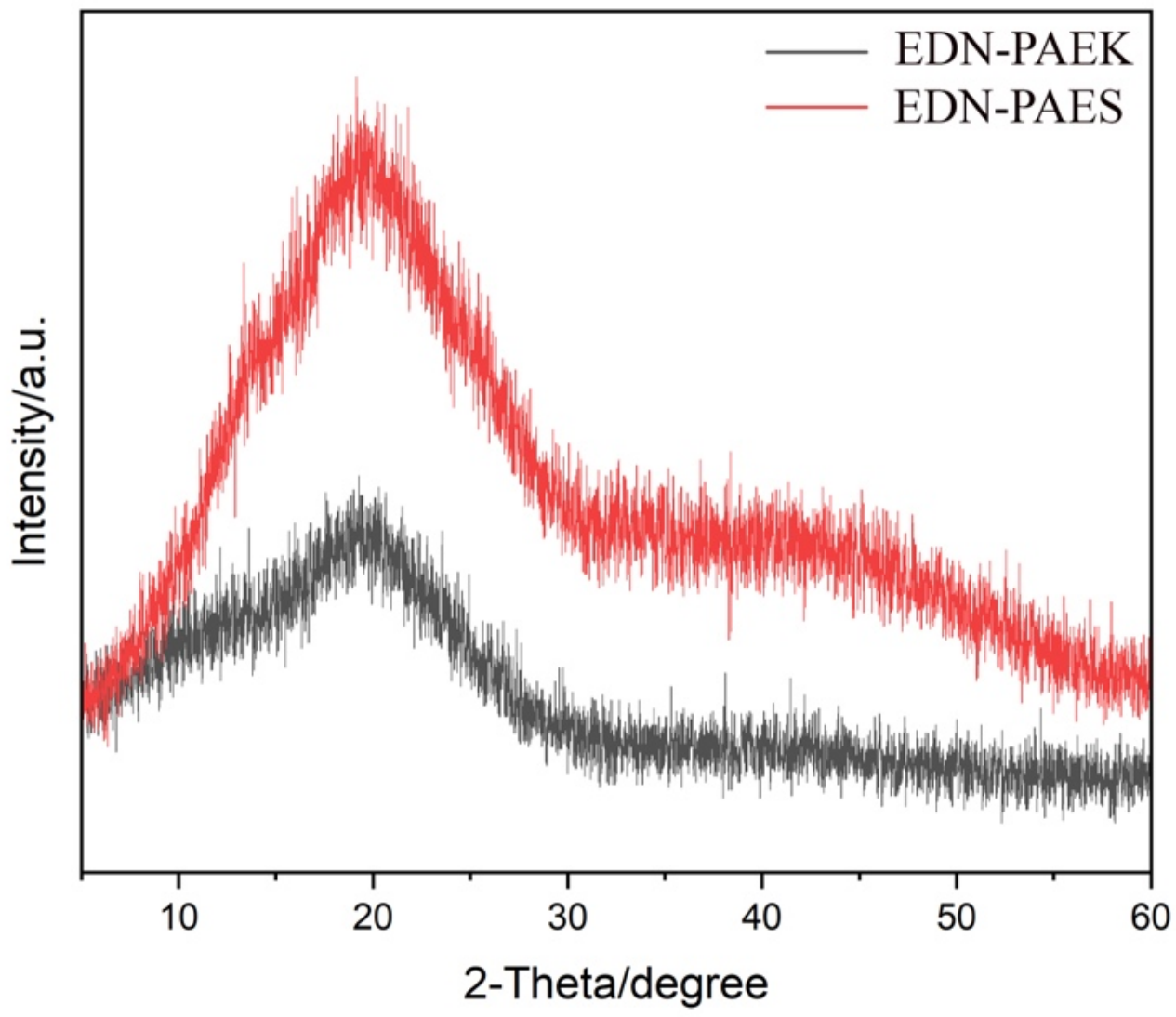

ESI Figure 6. Powder XRD analysis of EDN-PAEK and EDN-PAES.

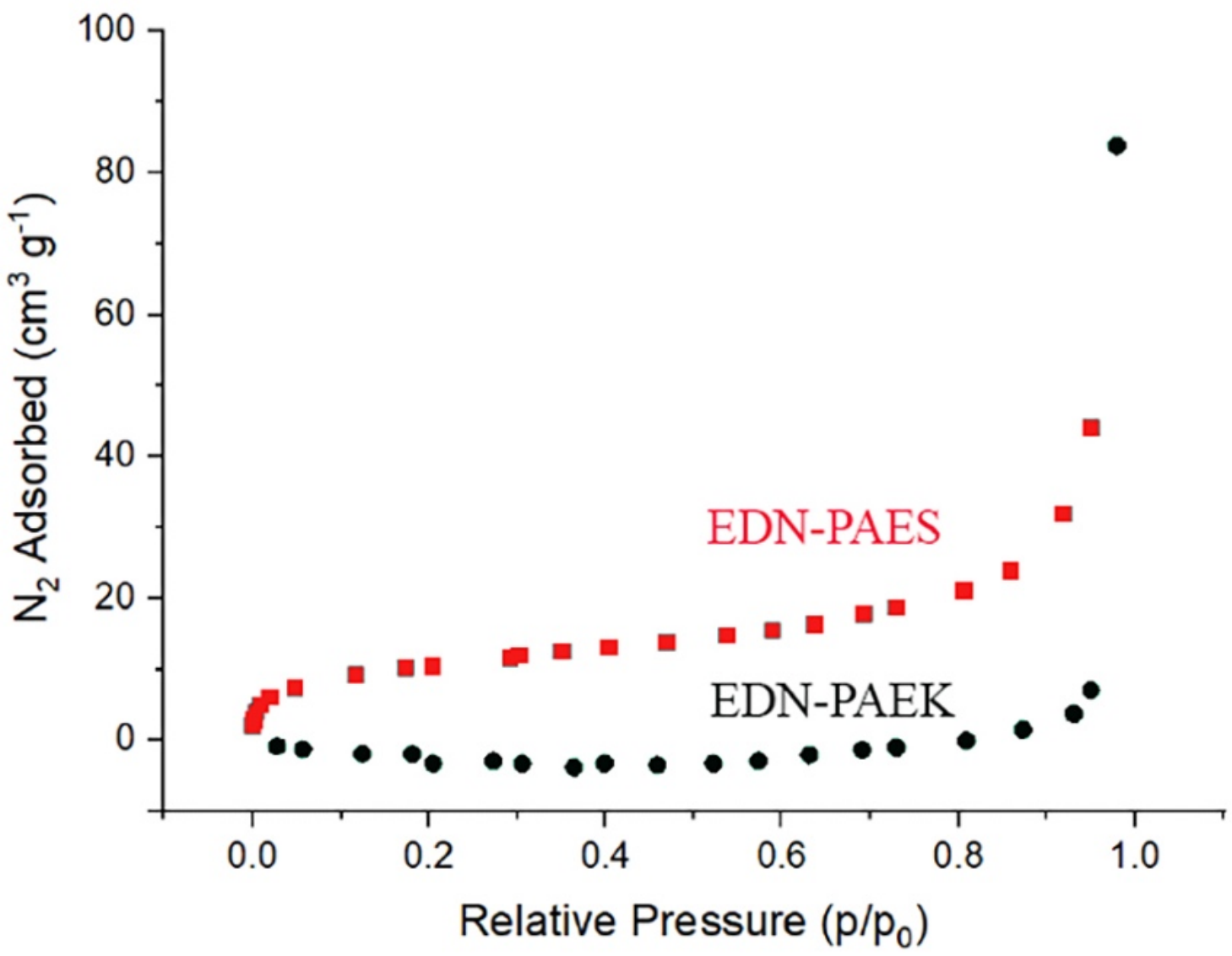

ESI Figure 7. N_2 adsorption isotherms (77 K) of EDN-PAEK and EDN-PAES.

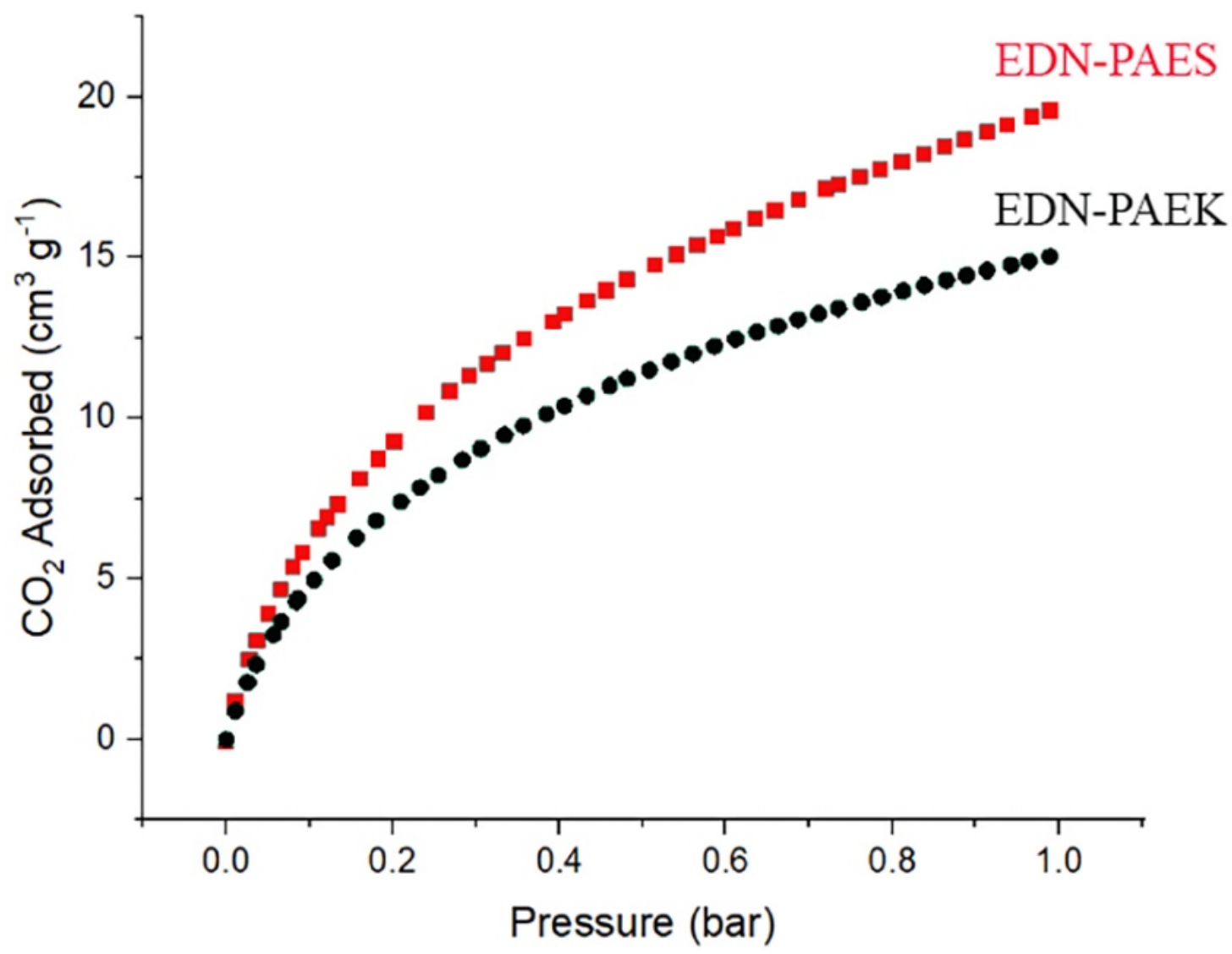

ESI Figure 8. CO_2 adsorption isotherms (273 K) of EDN-PAEK and EDN-PAES.

1.3 Membrane preparation

Membranes were prepared by casting from $CHCl_3$ solution (2 wt.%) into a flat glass Petri dish and allowing the solvent to evaporate slowly under ambient temperature. Free-standing membranes with thickness around 60 μm were peeled off from the Petri dish. The membranes were soaked in methanol for 24 h to remove the residual casting solvent and then dried for 24 h in air prior to the gas permeability measurements.

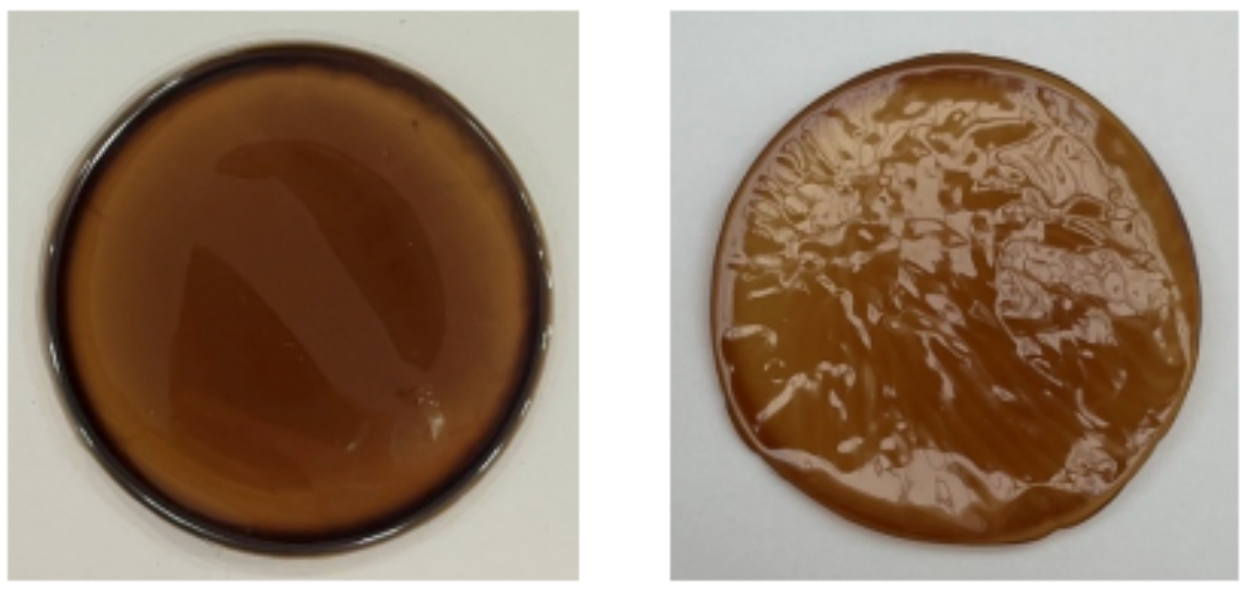

ESI Figure 9. Self-standing films of EDN-PAEK and EDN-PAES cast from $CHCl_3$ solutions.

2. Single-gas permeation measurements

Single-gas permeation tests were carried out at 35 °C and a feed pressure of 1 bar using a fixed-volume pressure-increase apparatus developed and constructed by EESR based on the HZG design (Geesthacht, Germany). The permeability (P) and diffusion (D) coefficients were determined using the time-lag method, while the solubility coefficient (S) was obtained indirectly from their ratio S = P / D. The ideal selectivities were calculated as the ratios of the permeability coefficients of the respective gas pairs.[1, 2]

Table SI1 Gas diffusion coefficients *D* (10^{-12} m^2s^{-1}) and diffusivity selectivities (D_A/D_B) measured at 35 °C and 1 bar after MeOH treatment and after 120 days of aging.

	Diffusivity coefficient						Diffusivity selectivity					
	N_2	O_2	CO_2	CH_4	H_2	He	CO_2/CH_4	CO_2/N_2	O_2/N_2	H_2/N_2	H_2/CH_4	He/N_2
EDN-PAEK	0.36	2.35	0.60	0.08	166	930	7.5	1.67	6.5	461	2076	2583
(120 d)	0.25	1.45	0.42	0.04	115	409	10.5	1.68	5.8	460	2875	1635
EDN-PAES	0.73	4.31	1.12	0.14	244	1401	8.0	1.53	5.9	334	1741	1919
(120 d)	0.55	2.58	0.84	0.05	206	666	16.8	1.52	6.7	374	4120	1210

Table SI2 Gas solubility coefficients *S* (cm^3_{STP} cm^{-3} bar^{-1}) and solubility selectivities (S_A/S_B) measured at 35 °C and 1 bar.

	Solubility coefficient						Solubility selectivity					
	N_2	O_2	CO_2	CH_4	H_2	He	CO_2/CH_4	CO_2/N_2	O_2/N_2	H_2/N_2	H_2/CH_4	He/N_2
EDN-PAEK	0.88	0.98	16.66	2.63	0.16	0.02	6.3	18.9	1.1	0.18	0.06	0.02
(120 d)	0.77	1.03	17.2	4.69	0.17	0.04	3.7	22.3	1.33	0.22	0.04	0.05
EDN-PAES	0.77	0.79	17.30	3.64	0.16	0.02	4.8	22.5	1.0	0.20	0.04	0.03
(120 d)	0.57	0.88	16.3	6.66	0.15	0.04	2.45	28.6	1.54	0.26	0.003	0.07

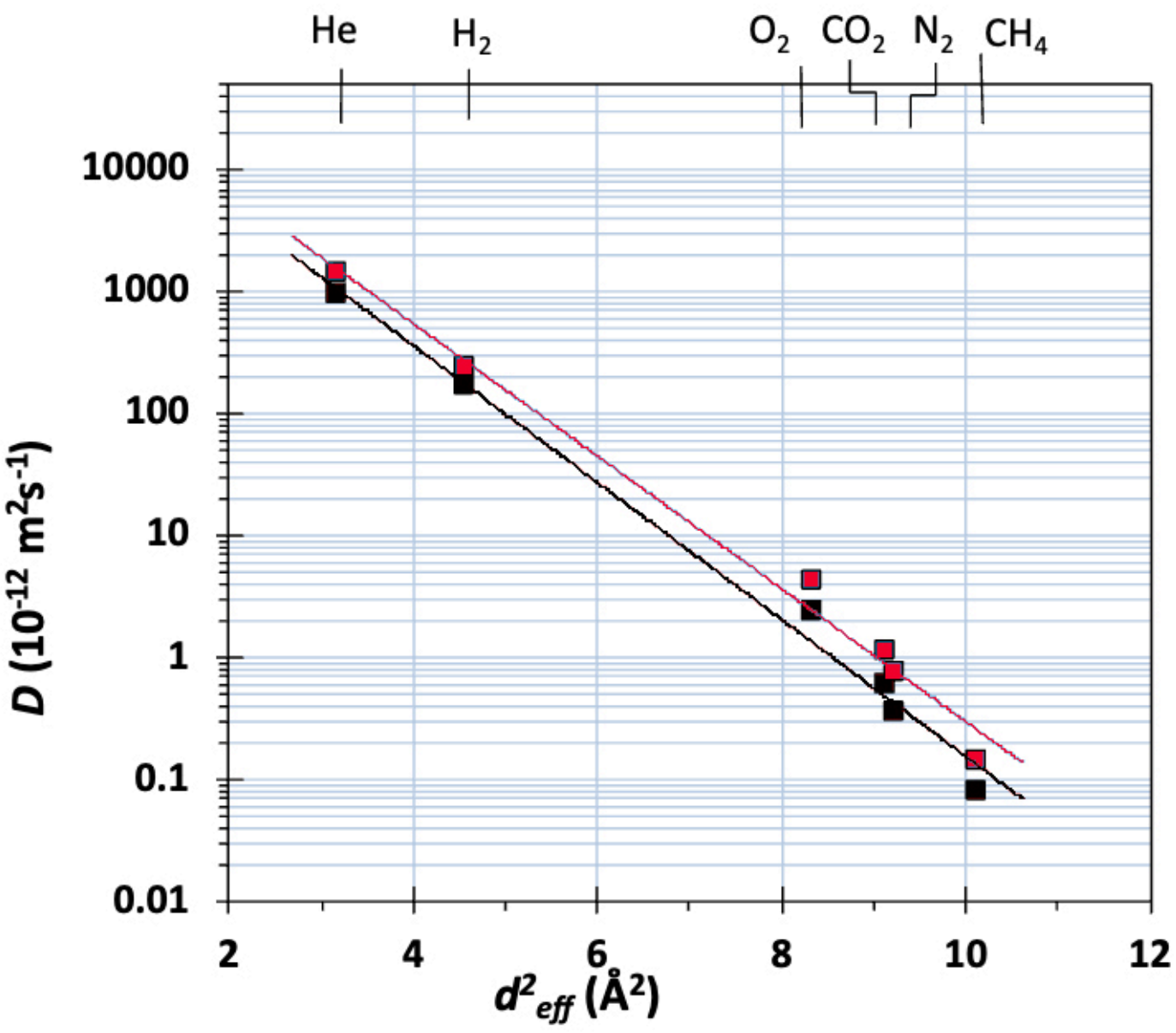

ESI Figure 10. Correlation of the diffusion coefficient within the films of EDN-PAEK (■) and EDN-PEAS (■)with the square of the Teplyakov-Meares gas diameter.[3]

References.

1. M. Monteleone, A. Fuoco, E. Esposito, I. Rose, J. Chen, B. Comesaña-Gándara, C. G. Bezzu, M. Carta, N. B. McKeown, M. G. Shalygin, V. V. Teplyakov and J. C. Jansen, *J. Membr. Sci.*, 2022, **648**, 120356.
2. A. Fuoco, M. Monteleone, E. Esposito, R. Bruno, J. Ferrando-Soria, E. Pardo, D. Armentano and J. C. Jansen, *Computation*, 2020, **8**, 28.
3. A. Fuoco, C. Rizzuto, E. Tocci, M. Monteleone, E. Esposito, P. M. Budd, M. Carta, B. Comesaña-Gándara, N. B. McKeown and J. C. Jansen, *J. Mater. Chem. A*, 2019, **7**, 20121-20126.
